# Enhanced Light Absorption by Facile Patterning of Nano-Grating on Mesoporous TiO_2_ Photoelectrode for Cesium Lead Halide Perovskite Solar Cells

**DOI:** 10.3390/nano11051233

**Published:** 2021-05-07

**Authors:** Kang-Pil Kim, Wook Hyun Kim, Soo Min Kwon, Jun Yong Kim, Yun Seon Do, Sungho Woo

**Affiliations:** 1 Division of Energy Technology, Daegu Gyeongbuk Institute of Science and Technology (DGIST), Daegu 42988, Korea; kkp@dgist.ac.kr (K.-P.K.); kwh1980@dgist.ac.kr (W.H.K.); soomin3542@dgist.ac.kr (S.M.K.); 2 School of Electronic and Electrical Engineering, Kyungpook National University, Daegu 41566, Korea; rhawns4567@knu.ac.kr

**Keywords:** CsPbIBr_2_, perovskite solar cell, mesoporous TiO_2_, nanoimprinting, grating nanopattern, increased light absorption

## Abstract

CsPbIBr_2_, a cesium-based all-inorganic halide perovskite (CsPe), is a very promising alternative material to mainstream organic–inorganic hybrid halide perovskite (HPe) materials owing to its exceptional moisture stability, thermal stability, and light stability. However, because of the wide band gap (2.05 eV) of CsPbIBr_2_, it has a low power conversion efficiency (*PCE*), which hinders its application in highly efficient solar cells. In this study, a facile nanoimprinted one-dimensional grating nanopattern (1D GNP) formation on mesoporous TiO_2_ (mp-TiO_2_) photoelectrodes was introduced to improve the effective light utilization and enhance the performance of CsPbIBr_2_ perovskite solar cells (PSCs). The 1D GNP structure on the mp-TiO_2_ layer increases the light absorption efficiency by diffracting the unabsorbed light into the active mp-TiO_2_ and CsPbIBr_2_ layers as well as increasing the charge separation and collection due to the extended interfacial contact area between the mp-TiO_2_ and CsPbIBr_2_ layers. Consequently, both the current density (*J_SC_*) and the fill factor (*FF*) of the fabricated cells improved, leading to over a 20% enhancement in the solar cell’s *PCE*. Thus, this periodic grating structure, fabricated by simple nanoimprinting, could play an important role in the large-scale production of highly efficient and cost-effective Cs-based PSCs.

## 1. Introduction

The power conversion efficiency (*PCE*) of organic–inorganic hybrid halide perovskite (HPe) solar cells has improved dramatically over the last ten years, rising from 3.81% in 2009 to 25.5% in 2021 [1,2]. This remarkable progress is mainly attributed to the outstanding properties of HPe cells, which include strong light absorption in the visible region, high charge carrier mobility, and long charge diffusion length [1,3]. HPe has a typical ABX_3_ structure, where A is an organic cation (mainly CH_3_NH_3_^+^ (MA^+^) and/or NH_2_CHNH_2_^+^ (FA^+^)), B is an inorganic cation (Pb^2+^, Ge^2+^, Sn^2+^), and X is a halogen anion (I^−^, Br^−^, Cl^−^). Although HPe cells can achieve a very high *PCE*, their thermal, light, and moisture stabilities are not sufficient for device commercialization because of the intrinsically volatile and hygroscopic properties of organic cation moieties, such as MA^+^ or FA^+^ [4].

To overcome the limitations of HPe, several methods have been developed, including metal doping in the perovskite phase [5], interface/surface treatment [6], device encapsulation [7], and employing an all-inorganic perovskite composition [8,9,10]. Among these methods, the most used strategy is to replace the volatile organic group with non-volatile inorganic cesium (Cs) cations to make a Cs-based all-inorganic perovskite (CsPe) light absorption layer. For example, CsPe-based (CsPbX_3_, where X = I, Br, Cl, or their mixture) perovskite solar cells (PSCs) have shown remarkable stability under 80% humidity or 100 °C heating conditions [11,12]. The efficiency of CsPbX_3_ is closely related to its halide composition, which leads to a change in its crystal structure, band gap, and humidity tolerance. The currently studied inorganic perovskite compositions are mainly CsPbI_3_, CsPbI_2_Br, CsPbIBr_2_, and CsPbBr_3_, based on the type of halide and its ratio. Among these all-inorganic Cs-based perovskites, α-phase CsPbI_3_ is the most widely investigated perovskite owing to its narrow band gap (E_g_ = 1.73 eV), suitability for absorbing visible light, and excellent efficiency of 17.06%. However, black α-phase CsPbI_3_ suffers from severe instability and easily degrades to a non-perovskite yellow δ-phase CsPbI_3_ (E_g_ = 2.82 eV), even at room temperature. These are the major issues that delimit the fabrication of solar cell devices using CsPbI_3_ [13,14]. On the other hand, the CsPbBr_3_ PSCs, all containing bromide CsPe, have shown exceptional stability, even without any passivation. However, a CsPbBr_3_ layer has a very narrow light absorption range of only up to 540 nm owing to its wide band gap (E_g_ = 2.3 eV), which results in a lower *PCE* [15]. Therefore, exchanging some iodide ions with bromide ions in CsPe to form a mixed-halide CsPe (CsPbI_3−x_Br_x_) could be a good alternative for improving the phase stability. CsPbI_2_Br, a mixed-halide CsPe, has a slightly narrow band gap of 1.92 eV and shows better stability than CsPbI_3_ does; however, for CsPbI_2_Br, overcoming the stability issue is still a challenge [16,17]. Finally, CsPbIBr_2_ (another mixed-halide CsPe) has been considered as the best candidate among all the possible Cs-based inorganic perovskites because it has an acceptable band gap of 2.05 eV and possesses a superior phase stability in air. Moreover, CsPbIBr_2_ has a typical bright red color that is suitable for applications in tandem devices and colorful smart photovoltaic windows [18,19]. Over the years, the *PCE* of CsPbIBr_2_ has been improved from 4.7% to the recent highest efficiency of 10.88% by introducing various strategies, such as the optimization of the fabrication process to obtain a better film quality, interface/surface engineering, and elemental impurity doping for energy-level control and good charge carrier transport [19,20,21,22,23]. Although the cell performance of CsPbIBr_2_ is significantly improved by these effective modifications, its *PCE* is still constrained compared to that of the organic–inorganic hybrid PSCs. This limited *PCE* is caused by a poor light absorption wavelength edge of 600 nm due to its intrinsic wide band gap. To address this limited light absorption characteristic, several photon management techniques, such as utilization of the surface plasmonic effect, anti-reflection schemes, and periodic gratings, have been applied and studied to increase the time of residence of the targeted photons in the photoactive layer [24,25,26,27,28]. Considering the results of these previous studies, we can conclude that a promising approach to improve the light absorption of a wide band gap CsPbIBr_2_ (without increasing its thickness) is to diffract the incident light and maximize the light dwelling time in the CsPbIBr_2_ layer by employing a periodic grating.

In this study, we introduced a one-dimensional grating nanopattern (1D GNP) over a mesoporous TiO_2_ (mp-TiO_2_) layer using solvent-assisted nanoimprinting and applied it to CsPbIBr_2_ PSCs to increase their light harvesting as well as their charge collection. As a result, the device with a flat mp-TiO_2_ layer exhibited a *PCE* of 3.5%, while the device with a 1D GNP mp-TiO_2_ layer achieved a *PCE* of 4.3%, which is an ~23% improvement in the *PCE* due to the increase in the photogenerated current density (*J_SC_*) and fill factor (*FF*) of the CsPbIBr_2_ PSCs. In addition, this periodic nano-grating process can be applicable for mass production at a low cost, as it allows high reproducibility with reusable masks and molds.

## 2. Materials and Methods

### 2.1. Materials Used and Nanopatterned PDMS Mold Preparation

CsI (99.9%), PbBr_2_ (99.999%), dimethyl sulfoxide (DMSO, anhydrous, >99.9%), chlorobenzene (CB, anhydrous, 99.8%), and 1H,1H,2H,2H-Perfluorooctyl-trichlorosilane (PFOTCS, 97%) were purchased from Sigma-Aldrich (St. Louis, MO, USA). TiO_2_ solutions, SC-BT060 and SC-HT040 (anatase structure, particle size 50 nm), were purchased from Sharechem Co. (Hwaseong, South Korea) to fabricate the compact TiO_2_ (c-TiO_2_) layer and the mp-TiO_2_ layer, respectively. Poly[[4,8-bis[(2-ethylhexyl)oxy]benzo[1,2-b:4,5-b’]dithiophene-2,6-diyl][3-fluoro-2-[(2-ethylhexyl)carbonyl]thieno[3,4-b]thiophenediyl]] (PTB7, average molecular weight of 116 kDa, polydispersity index of 2.5) was purchased from 1-Material Inc (Dorval, QC, Canada). All chemicals were used in the form as delivered by the manufacturer without further purification.

To prepare a 1D GNP prepatterned polydimethylsiloxane (PDMS) mold, the silicon (Si) master (grating period of 605 nm, grating depth of 190 nm; LightSmyth Technologies Inc. (Eugene, OR, USA)) was set on a thin plate. A few drops of PFOTCS near the Si master were used to make the Si master surface hydrophobic to facilitate easy removal of the Si master from the PDMS mold. Next, the PDMS pre-polymer was obtained by mixing a Sylgard 184 elastomer kit (with a 10:1 weight ratio of elastomer base to curing agent, Dow Corning (Midland, MI, USA)) for 30 min and letting it degas for 1 h in a vacuum oven. Then, the PDMS pre-polymer was poured onto the Si master and cured at 100 °C for 40 min. The cured PDMS mold was detached from the Si master and used for the nanopatterning of the mp-TiO_2_ layer.

### 2.2. Device Fabrication

**Figure 1:**
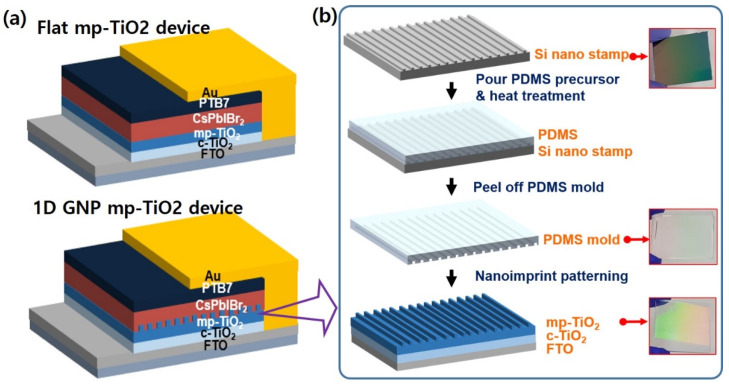
Schematics of (**a**) the device structure of CsPbIBr_2_ solar cells with flat mp-TiO_2_ and 1D GNP mp-TiO_2_, and (**b**) the fabrication process for 1D GNP mp-TiO_2_.

We fabricated CsPbIBr_2_ solar cells with an FTO/c-TiO_2_ (30 nm)/mp-TiO_2_ (330 nm)/CsPbIBr_2_ (300 nm)/PTB7 (40 nm)/Au (70 nm) structure, where PTB7 was used as a hole transport layer (HTL) (see Figure 1a). After cleaning the FTO substrate, a 30-nanometer c-TiO_2_ layer was spin-coated using an SC-BT060 solution at 3000 rpm for 30 s and annealed at 500 °C for 30 min. Then, to deposit the flat mp-TiO_2_ layer, the SC-HT040 paste was spin-coated and annealed on the bl-TiO_2_/FTO substrate at 3000 rpm for 30 s and at 500 °C for 30 min, respectively. For the 1D GNP mp-TiO_2_ layer, the PDMS mold was immediately placed onto the spin-coated mp-TiO_2_ paste with appropriate pressure to apply a nanoimprinting process before the paste surface dried. After complete surface contact between the PDMS mold and the mp-TiO_2_ paste, a heat treatment at 125 °C for 5 min was performed to remove the residual solvent. Next, the nanopatterned mp-TiO_2_ was separated from the PDMS mold and annealed at 500 °C for 30 min (see Figure 1b).

To prepare the CsPbIBr_2_ layer, a precursor solution (1 M solution of CsI and PbBr_2_ in DMSO) was spin-coated on the flat or 1D GNP mp-TiO_2_ layer at 2500 rpm for 60 s and heated at 160 °C for 20 min. Subsequently, PTB7 solution (5 mg/1 mL CB) was spin-coated on the CsPbIBr_2_ layer at 3000 rpm for 60 s and dried on a hot plate at 70 °C for 30 min in air. Finally, a 70-nanometer Au layer was deposited to make counter electrodes using a thermal evaporator under a high vacuum pressure of 5.0 × 10^−6^ Torr. The active area of the fabricated devices was 0.16 cm^2^.

### 2.3. Characterization and Analysis

The cross-sectional and top-view images of the mp-TiO_2_ layer were obtained using field emission scanning electron microscopy (FE-SEM, S-4800, Hitachi, Tokyo, Japan). The transmission, reflection, and absorption properties of mp-TiO_2_ were characterized using UV–Vis spectrometry (Lambda 750, Perkin Elmer, Waltham, MA, USA). A multi-purpose X-ray diffraction (XRD) system (Empyrean, PANalytical, Almelo, The Netherlands) was used to determine the structure of the CsPbIBr_2_ perovskite layer. The current density–voltage (*J*–*V*) characteristics under light and dark conditions were measured using a solar cell measurement system equipped with a Keithley-2400 source measure unit and a solar simulator (91192, Newport, Irvine, CA, USA). The light intensity of the illumination was set to AM1.5G (100 mW/cm^2^) and monitored using a radiant power meter (70260, Oriel). The external quantum efficiency (EQE) spectrum was measured using a QuantX-300 system (Newport). The series resistance (*R_S_*) and shunt resistance (*R_SH_*) of the devices were extracted from the slope of the light *J–V* curves near the open-circuit voltage (*V_OC_*) and *J_SC_* regions, respectively.

To investigate the optical absorption in the CsPbIBr_2_ active layer, the solar cells were analyzed using the three-dimensional (3D) finite difference time-domain (FDTD) method (FDTD Solution, Lumerical Inc., Vancouver, BC, Canada). In the optical simulation, the solar cells were composed of glass/FTO/c-TiO_2_/mp-TiO_2_ (flat and 1D GNP)/CsPbIBr_2_/PTB7/Au. A plane wave with a wavelength range of 300 to 700 nm was used as an incident light source. A frequency-domain field and power monitor was set up to evaluate the total absorbed power and the spatial profile of the optical absorption per unit volume of the CsPbIBr_2_ active layer.

## 3. Results and Discussion

The mp-TiO_2_ with a 1D GNP structure exhibits a rainbow-colored visual effect due to the optical interference caused by the grating nanopattern (see the bottom inset of Figure 1b).

**Figure 2:**
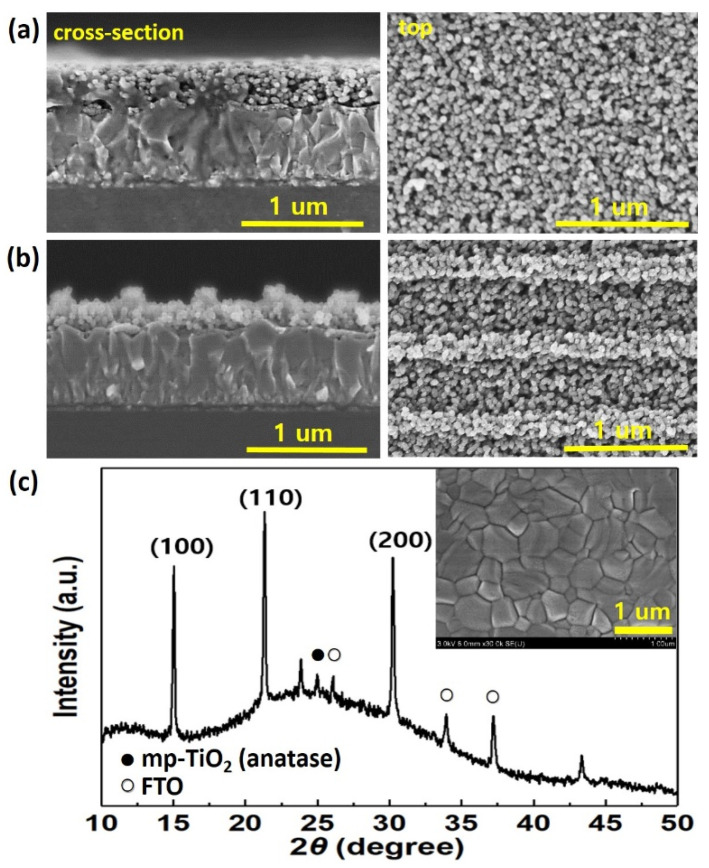
(**a**) Cross-section and top-view SEM images of the flat mp-TiO_2_ and (**b**) the 1D GNP mp-TiO_2_. (**c**) XRD data for the CsPbIBr_2_ layer (the inset shows the surface morphology of the CsPbIBr_2_ layer). The solid circle and open circle denote the peaks from the mp-TiO_2_ (anatase) and FTO substrates, respectively.

Figure 2a,b show the SEM images of the mp-TiO_2_ layer, with and without a 1D GNP structure, respectively. The cross-sectional and top-view images of the 1D GNP mp-TiO_2_ show a clear periodic pattern over the entire large area without fragments or cracks. The period, width, and height of the 1D GNP mp-TiO_2_ are about 610, 250, and 100 nm, respectively. XRD measurements were performed to reveal the formation of the CsPbIBr_2_ perovskite layer (Figure 2c). The typical diffraction peaks in the XRD patterns were matched to α-phase CsPbIBr_2_ and were found to be consistent with those in previous reports. The three dominant diffraction peaks at 15.02°, 21.27°, and 30.15° that are indexed to the (100), (110), and (200) planes, respectively, show good crystallinity and morphology of the CsPbIBr_2_ perovskite layer, which are further confirmed by the SEM image in the inset of Figure 2c [20,29,30,31]. We can also see the XRD peak at 25.2° from the (101) plane of the anatase TiO_2_ structure, as we used a TiO_2_ nanoparticle solution (crystal structure: anatase; average size: 50 nm) to fabricate an mp-TiO_2_ layer. The anatase TiO_2_ has been generally accepted as an electron transport layer of solar cells because of its low cost, transparency, and higher conduction band level for a fast electron transport and high *V_OC_* [32,33].

**Figure 3:**
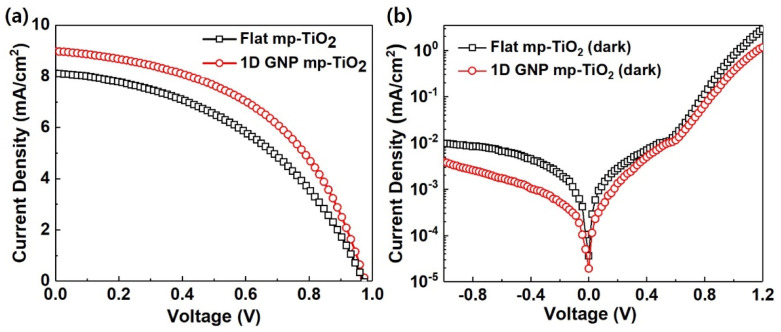
Current density–voltage (*J–V*) properties of CsPbIBr_2_ solar cells with a flat mp-TiO_2_ and a 1D GNP mp-TiO_2_ under (**a**) one sun illumination and (**b**) dark conditions.

Figure 3a and Table 1 represent the light *J–V* properties for the solar cells with and without a 1D GNP pattern under AM 1.5G (100 mW/cm^2^). The efficiency of the CsPbIBr_2_ solar cell was improved by the introduction of a 1D GNP onto the mp-TiO_2_ layer. The flat mp-TiO_2_ device exhibited a *V_OC_* of 0.971 V, a *J_SC_* of 7.98 mA/cm^2^, an *FF* of 45.17%, and a *PCE* of 3.5%. However, in the 1D GNP mp-TiO_2_ device, all these parameters increased. The *V_OC_*, *J_SC_*, and *FF* increased to 0.975 V, 8.88 mA/cm^2^, and 49.73%, respectively, leading to a *PCE* of 4.31%. The introduction of a 1D GNP pattern resulted in a *PCE* enhancement of ~23%, primarily due to the improvement in the *J_SC_*. (Here, we note that although there is a large difference in the *PCE* compared with the recently reported best efficiency of 10.88% [18,22], our device efficiency of ca. 4% is reasonable because the devices were made via a very basic one-step coating process without using any improved methods, such as anti-solvent dropping, interface treatment, impurity doping, etc.)

**Figure 4:**
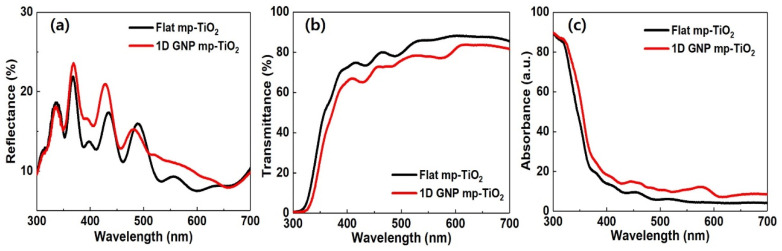
(**a**) Reflectance, (**b**) transmittance, and (**c**) absorbance spectra of the flat mp-TiO_2_ and the 1D GNP mp-TiO_2_ layers.

To understand the working principle for these improvements, we further investigated the optical and electrical properties of the devices. First, the reflectance, transmittance, and absorbance of the glass/FTO/c-TiO_2_/mp-TiO_2_ samples were measured using a UV–Vis spectrometer to study the optical effect of a 1D GNP on the mp-TiO_2_, where the beam was incident on the glass side. The measured reflectance of the mp-TiO_2_ with a 1D GNP (average ≈13.8%) was higher than that of the flat mp-TiO_2_ (average ≈12.3%), as shown in Figure 4a. In contrast, the transmittance of the mp-TiO_2_ with a 1D GNP (average ≈ 62.2%) was lower than that of the flat mp-TiO_2_ (average ≈ 69.1%). Therefore, the 1D GNP mp-TiO_2_ can absorb more light than the flat one over a broad range of wavelengths (see Figure 4b,c) [34,35]. This is attributed to the increased optical path length and light trapping effect that diffracts the unabsorbed light back into the mp-TiO_2_ photoelectrode at an oblique angle, which can further result in enhanced photon absorption in the CsPbIBr_2_ active layer, as depicted in the FDTD simulation results.

**Figure 5:**
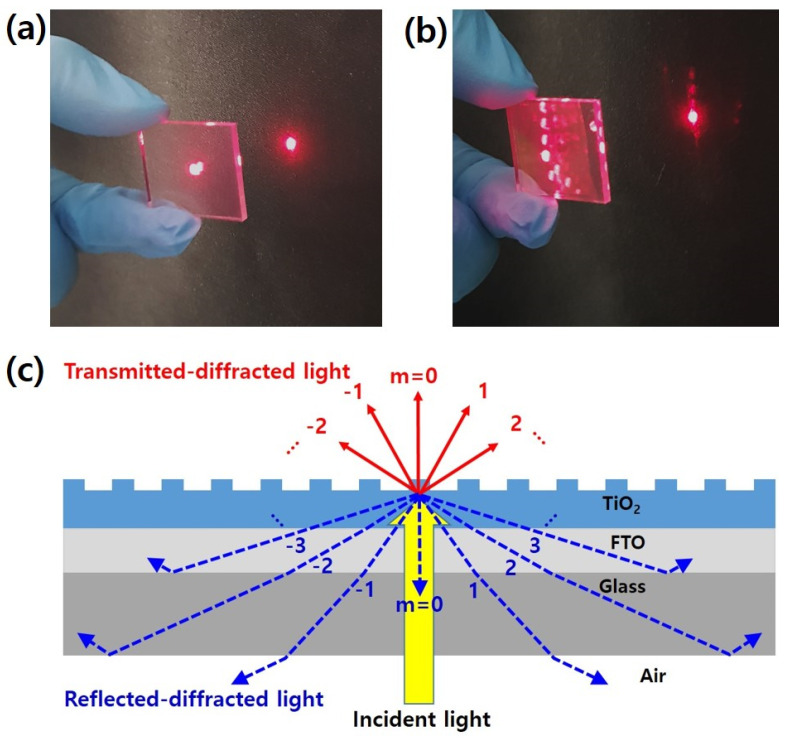
Digital camera images of the diffracted light from (**a**) a flat mp-TiO_2_ and (**b**) a 1D GNP mp-TiO_2_. (**c**) Schematic illustration of grating diffraction.

Compared to the flat mp-TiO_2_, we can see the obvious light diffraction effect of the 1D GNP mp-TiO_2_ from the corresponding digital camera images in Figure 5.

As illustrated in Figure 5c, the incident light is diffracted into multiple orders of transmitted and reflected light (m = …, −2, –1, 0, 1, 2, …) toward different directions at the grating interface [36]. Figure 5a,b show the light diffracted and reflected by the flat mp-TiO_2_ and the 1D GNP mp-TiO_2_, respectively, using a He–Ne laser (632.8 nm). In Figure 5a,b, we can observe the reflected light on the glass surface and the diffracted light on the front black screen in the case of the 1D GNP mp-TiO_2_, whereas the flat mp-TiO_2_ shows only a strong transmitted light without the diffracted and reflected light. Additionally, the zeroth-order transmitted light of the 1D GNP mp-TiO_2_ is weaker than that of the flat mp-TiO_2_, which corresponds to the reflectance and transmittance spectra shown in Figure 4a,b. Furthermore, if the angle of the reflected–diffracted light is larger than the critical angle for the total internal reflection because of the slightly decreased refractive index values from the TiO_2_ layer to air (i.e., n_TiO2_ (≈2.1) > n_FTO_ (≈1.9) > n_Glass_ (≈1.5) > n_Air_ (=1.0), where these refractive index values at 550 nm were estimated from previous reports [37,38]), then the light can be reflected back to the mp-TiO_2_ and perovskite layers, leading to an additional absorption, as illustrated in Figure 5c.

**Figure 6:**
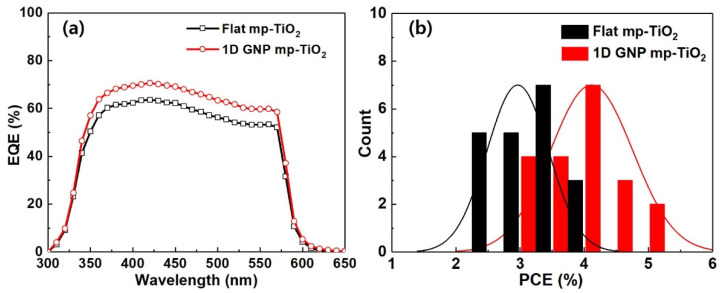
(**a**) External quantum efficiency (EQE) spectra and (**b**) *PCE* distribution histogram of the CsPbIBr_2_ PSCs with and without a 1D GNP structure in the mp-TiO_2_ for 20 devices.

Next, we performed EQE measurements to confirm whether the current enhancement was the main factor increasing the device’s performance in the 1D GNP mp-TiO_2_ structure. As can be seen in Figure 6a, the EQE was improved across the entire spectral range, and the enhancement was distinguishable in the wavelength range from 330 to 570 nm. The average EQE in the measured spectral range increased by ~12.1% when accompanied by the 1D GNP structure. These EQE results indicate that a 1D GNP structure can help to reuse the diffracted light to generate more charge carriers by increasing the optical path length and light trapping.

The device with the 1D GNP mp-TiO_2_ showed a higher *FF* in comparison to the control device with the flat mp-TiO_2_. We calculated the internal resistance of the devices (i.e., *R_S_* and *R_SH_*) from the light *J–V* curve and also measured the dark *J–V* characteristic to study the effect of a 1D GNP on device resistance. The higher *FF* of the 1D GNP may have been caused by the reduced interface resistance due to the increase in the contact area between the 1D GNP mp-TiO_2_ and the CsPbIBr_2_ active layer, as shown in the dark *J–V* curves in Figure 3b. The decreased *R_S_* and increased *R_SH_* indicate a better charge transport that could be another factor causing the observed high *FF* in the devices with a 1D GNP mp-TiO_2_ [37,39].

To compare the performance enhancement exhibited by the 1D nanostructures, we fabricated and measured 20 perovskite solar cells, with and without the 1D GNP mp-TiO_2_ layer. Figure 6b shows the histograms of *PCE* distributions for each type of solar cell. We can observe a consistent and reproducible *PCE* improvement trend in the case of the 1D GNP mp-TiO_2_ structure.

**Figure 7:**
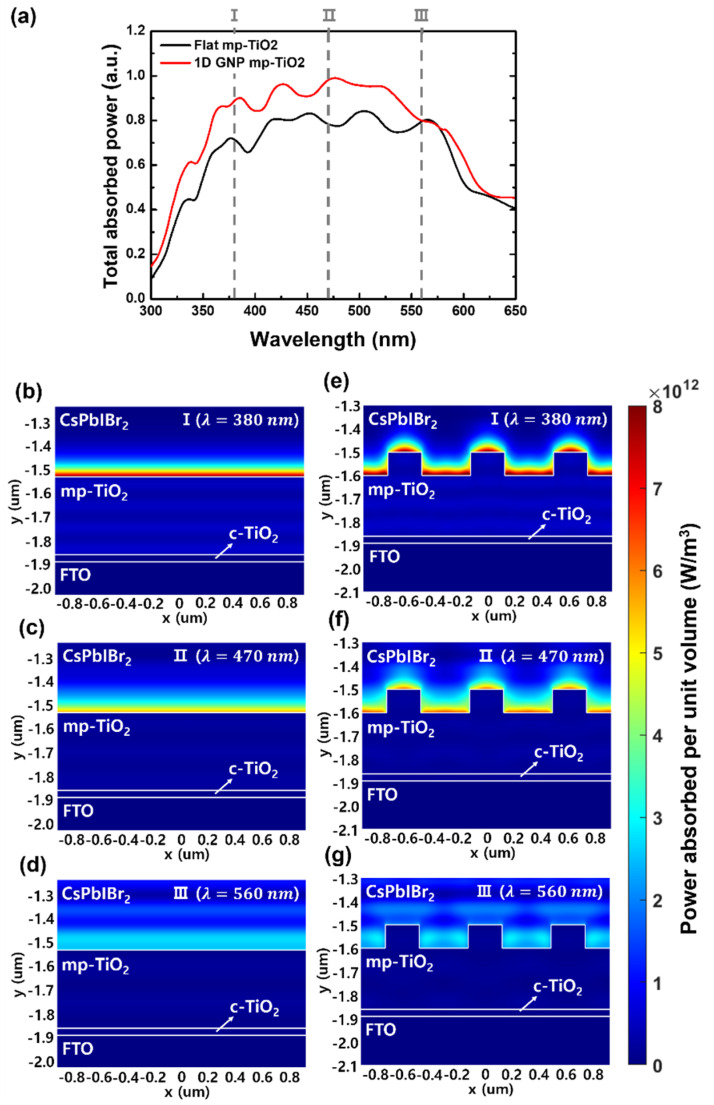
Calculated results of the solar cells with flat and 1D GNP mp-TiO_2_ layers. (**a**) Spectral response of total absorbed power in the CsPbIBr_2_ active layer. (**b**–**g**) Spatial profiles of power absorbed per unit volume with respect to the xy-plane at the three different wavelengths (380, 470, and 560 nm): (**b**–**d**) case of the flat mp-TiO_2_ layer; (**e**–**g**) case of the 1D GNP mp-TiO_2_ layer.

Finally, we performed numerical simulations using the FDTD method to investigate the effects of the diffraction grating in the fabricated solar cell. Figure 7 shows the optical characteristics of the total device structure illustrated in Figure 1, especially for absorbed power. The spectral response of the average absorbed power in the CsPbIBr_2_ layer with a flat and a 1D GNP mp-TiO_2_ is shown in Figure 7a. Considering the total spectral absorbed power, the device with the 1D GNP mp-TiO_2_ layer showed higher absorption in the active layer than that of the device with the flat mp-TiO_2_ layer. Although this enhancement factor is not exactly the same as that of the EQE results, the spectral trend corresponds with the spectral trend of the EQE of fabricated solar cells in Figure 6a. In the case of the absorbed power, the increase was distinguishable in the wavelength range from 330 to 560 nm. The average value of the absorbed power in the wavelength range from 300 to 650 nm was 0.760 in the 1D GNP structured device. This represents an increase of 17.1% compared to the flat device, which had a value of 0.649 for the average spectral absorbed power.

The spatial profiles of power absorbed per unit volume with respect to the xy-plane of the solar cells with a flat and a 1D GNP mp-TiO_2_ were analyzed to further evaluate the diffraction effects in a CsPbIBr_2_ layer. Figure 7b–d show the light absorption profiles of the flat mp-TiO_2_ device and Figure 7e–g show those of the 1D GNP mp-TiO_2_ structure at wavelengths of 380, 470, and 560 nm, respectively. The optical absorption for the solar cells with a flat mp-TiO_2_ layer occurs strongly at the interface between the mp-TiO_2_ layer and the CsPbIBr_2_ layer. Compared to the flat mp-TiO_2_, the amount of absorption in the CsPbIBr_2_ layer with a 1D GNP mp-TiO_2_ increased at the interface between the mp-TiO_2_ layer and the CsPbIBr_2_ layer by the diffraction grating effects at wavelengths of 380 and 470 nm, respectively. At the wavelength of 560 nm, the value of the total absorbed power at point III in Figure 7a appears almost the same because the absorbed power of the absorbing layer is similar to that in the spatial profile in Figure 7d,g.

It shows that the light is trapped inside the active layer by the increased optical path length and the total internal reflection when the light is diffracted at a specific wavelength. In addition, the diffracted light within an absorption layer improves the light harvesting at the interface between the mp-TiO_2_ layer and the CsPbIBr_2_ layer [27,28]. The fabricated 1D GNP mp-TiO_2_ layer was not completely square shaped, as shown in Figure 2b. In addition, the measured refractive index values were applied to the mp-TiO_2_ grating structure without porous media. The amount of absorption in the active layer was not exactly matched with the enhanced factor of the EQE in the experimental results owing to these imperfections. However, the overall trends correspond with each other, and it can be inferred that the diffraction grating enhances the absorption inside the active layer and consequently improves the power conversion efficiency of the solar cells. Moreover, because the grating pattern is formed with periodicity, it is advantageous for mass production compared to other nano patterns. For example, laser interference lithography contains the exposure process with the interference of a light source that can be formed by two-beam interference; thus, it does not require any masking components. The nanoimprinting technology used in this work shows high reproducibility with reusable masks [40,41].

## 4. Conclusions

We successfully introduced a 1D GNP structure onto mp-TiO_2_ and fabricated a high-performance CsPbIBr_2_ perovskite solar cell using a simple nanoimprinting method with a PDMS mold. It is evident from the UV–Vis spectra, EQE measurement data, and FDTD simulation data that the 1D GNP structure can diffract the incident light to increase the optical path length within the mp-TiO_2_ and active layers, thus leading to an enhancement in the light harvest of the devices over a broad wavelength range. In addition, the 1D GNP structure increases the *FF* by improving the electron extraction and suppressing the charge recombination due to the increased interfacial contact area between the mp-TiO_2_ and CsPbIBr_2_ layers, where excitons are separated and charge transfer occurs. As a result, the PSCs with the 1D GNP mp-TiO_2_ achieved a *PCE* of 4.31%, which is almost 23% higher than that of the flat mp-TiO_2_ PSCs (3.5%).

We believe that nanoimprinting a 1D GNP structure onto an mp-TiO_2_ photoelectrode, which optimizes light harvesting, can be an effective way to improve the photoelectric properties of various Cs-based perovskite applications, including solar cells, photodetectors, light-emitting diodes, and solar water-splitting devices.

## Figures and Tables

**Table 1:** Summary of device performance with a flat mp-TiO_2_ and a 1D GNP mp-TiO_2_ under AM 1.5 G conditions.

Device	*J_SC_* (mA/cm^2^)	*V_OC_* (V)	*FF* (%)	*PCE* (%)	*R_S_* (Ωcm^2^)	*R_SH_* (Ωcm^2^)
Flat mp-TiO_2_	7.98	0.971	45.17	3.50	37.21	1272.73
1D GNP mp-TiO_2_	8.88	0.975	49.73	4.31	25.98	1773.91

## Data Availability

Not applicable.
